# On the Role of Protonic Acid Sites in Cu Loaded FAU31 Zeolite as a Catalyst for the Catalytic Transformation of Furfural to Furan

**DOI:** 10.3390/molecules26072015

**Published:** 2021-04-01

**Authors:** Łukasz Kuterasiński, Małgorzata Smoliło-Utrata, Joanna Kaim, Wojciech Rojek, Jerzy Podobiński, Katarzyna Samson, Dorota Duraczyńska, Małgorzata Zimowska, Mariusz Gackowski, Dorota Rutkowska-Zbik

**Affiliations:** Jerzy Haber Institute of Catalysis and Surface Chemistry, Polish Academy of Sciences, Niezapominajek 8, 30-239 Krakow, Poland; ncsmolil@cyf-kr.edu.pl (M.S.-U.); joanna.miasik@ikifp.edu.pl (J.K.); wojciech.rojek@ikifp.edu.pl (W.R.); ncpodobi@cyf-kr.edu.pl (J.P.); ncsamson@cyf-kr.edu.pl (K.S.); ncduracz@cyf-kr.edu.pl (D.D.); nczimows@cyf-kr.edu.pl (M.Z.); ncgackow@cyf-kr.edu.pl (M.G.)

**Keywords:** faujasite, Brønsted acidity, copper, furan, furfural

## Abstract

The aim of the present paper is to study the speciation and the role of different active site types (copper species and Brønsted acid sites) in the direct synthesis of furan from furfural catalyzed by copper-exchanged FAU31 zeolite. Four series of samples were prepared by using different conditions of post-synthesis treatment, which exhibit none, one or two types of active sites. The catalysts were characterized by XRD, low-temperature sorption of nitrogen, SEM, H_2_-TPR, NMR and by means of IR spectroscopy with ammonia and CO sorption as probe molecules to assess the types of active sites. All catalyst underwent catalytic tests. The performed experiments allowed to propose the relation between the kind of active centers (Cu or Brønsted acid sites) and the type of detected products (2-metylfuran and furan) obtained in the studied reaction. It was found that the production of 2-methylfuran (in trace amounts) is determined by the presence of the redox-type centers, while the protonic acid sites are mainly responsible for the furan production and catalytic activity in the whole temperature range. All studied catalysts revealed very high susceptibility to coking due to polymerization of furfural.

## 1. Introduction

Furan is a heterocyclic organic compound whose molecule consists of a five membered aromatic ring formed by four carbon and one oxygen atoms. It is a precious precursor, mainly used in the production of chemicals such as α-acetylfuran, 2,2-difurylpropane, pyrrole derivatives and many other compounds [1].

Furan may be produced using 1,3-butadiene or furfural as substrates. The first option is a partial oxidation of 1,3-butadiene in the vapor phase at 500 °C over MoO_3_ and WO_3_ catalysts, nevertheless the obtained furan yields are very poor (10–15%) in spite of utilization of promoters such as Mo, Ni and Co [1].

**Figure 1:**
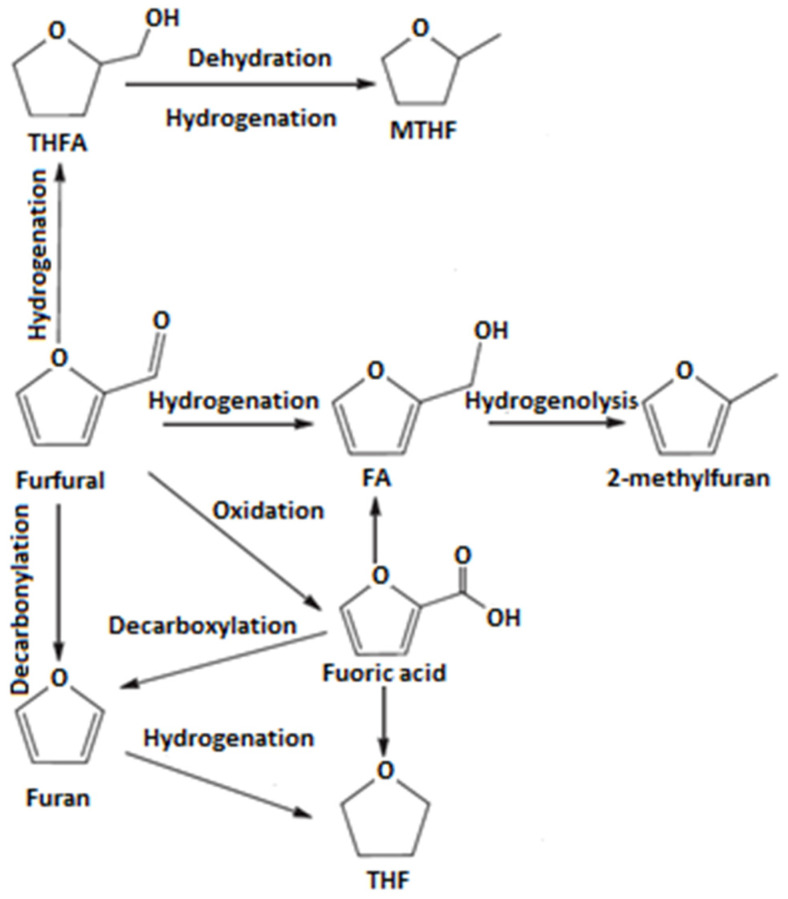
Possible pathways of hydrogenation of furfural based on data from [3].

Production from furfural, which is the second option, can proceed via different routes, employing different classes of catalysts. Originally, furan was produced from furfural via furoic acid as an intermediate (Cannizzaro reaction), followed by decarboxylation to produce furan [2]. More straightforward way is a direct decarbonylation of furfural with a release of a molecule of carbon monoxide (see Figure 1 [3]), the process that may be treated as a side reaction during the hydrogenation of furfural [3,4].

Most of reports describe the use of precious metals as catalysts. The decomposition of furfural into furan was investigated over Pd catalysts either in liquid or vapor phase in the temperature range of 150–250 °C [5,6]. Singh et al. [3,7] studied decarbonylation of furfural over Pd/C and Pd/Al_2_O_3_, out of which carbon was better carrier than Al_2_O_3_. It was found that the presence of hydrogen enhanced the furan yield and promoted the transport process of the reagents to/from the active sites in catalysts. Furan was also obtained by heating of furfural at 160 °C in the presence of 5% Pd supported on microporous carbon and potassium carbonate (furan yields over 98%) [3,8]. In turn, Bhogeswararao and Srinivas [9] used Pt/Al_2_O_3_ catalyst for which maximum furan yield was 50% at 240 °C.

Other, non-noble metal catalysts were tested as well. Wilson [3,10] described the production of furan from furfural over nickel gauze catalyst (yields > 50%). Decomposition of furfural into furan was also studied by Lejemble et al. [3,11] in the presence of steam over metal oxide catalysts, such as Zn/Fe or Zn/Mn chromite at 400 °C. High temperature may lead to the ring opening of furan towards heavy products and carbon deposit on the surface of catalyst. Hurd et al. [3,12] reported direct production of furan from furfural over fused alkali (with yields up to 60%) and the pyrolysis of furfural (yields ca.16.5%).

Next, production of furan from furfural was tested over systems containing acid sites such as present in zeolites (at 300–500 °C). Fanchiang and Lin [13] studied H-ZSM-5 and Zn/H-ZSM-5-based catalysts in a continuous fixed bed system. The reaction temperature, contact time and the presence of promoters were crucial for the manipulation of final products composition. At 300, 400 and 500 °C, furan yields were 16.5%, 30.6% and 19.5% respectively. Doping H-ZSM-5 with 1.5% Zn led to increase of furan yields at lower temperatures and disappearance of furan at 500 °C in comparison to the H-ZSM-5 system. For Zn-containing sample, furan yields were 30.7%, 45.0% and 0% at 300, 400 and 500 °C, respectively.

Finally, catalysts combining acid and metal active sites were also investigated. Seo and Chon [14] tested furfural hydrogenation in a fixed-bed stainless-steel tubular integral reactor at 160–350 °C in the presence of Pd-HY and Pd-LaY. The obtained furan yields were 50 and 38%, respectively. Taking into account that HY and LaY possess strong Brønsted acid sites [15,16], it was postulated that the presence of furan resulted from the interaction between polar carbonyl group of furfural and zeolitic acid sites. Pd probably activated the hydrogen [14].

Analysis of literature prompted us to take part in the discussion concerning the nature and contribution of various types of active centers, metallic “redox” type and acidic ones, in the mechanism of furfural to furan transformations. To do so, we have prepared samples containing FAU31 zeolite modified with copper species as the catalyst for the abovementioned reaction, which have one or two of the discussed active centers: Brønsted acid sites related to the zeolite structure as well as the “redox” type copper species (playing also a role of Lewis acid sites). We aimed at defining the role of particular active center in furfural to furan reaction by the systematic studies of catalytic activity of the prepared samples.

The chosen zeolite, HFAU31, is a commercial material with faujasite type structure after both ultrastabilization and partial dealumination (Si/Al = 31). Strength of acid sites present in FAU31 is higher in relation to the ones present in other zeolites, which are widely used in chemical industry like MFI, BEA or MOR [17].

Copper-containing zeolites are commonly used as active catalysts for numerous chemical processes, mostly “DeNOx”, but also oxidation, isomerization and dehydration [18,19,20,21,22,23,24,25,26,27], and thus, their properties were the subject on subject of very extensive studies: see, e.g., [28,29,30,31,32,33,34,35,36]. Copper active sites play an important role in activation of unsaturated bonds: double carbon-carbon bonds in alkenes, triple carbon-carbon bonds in ethyne, C=O bonds in acetone and formaldehyde as well as aromatic bonds in benzene [37,38,39,40]. Further, the IR and quantum mechanics studies [41,42] evidenced strong activation of hydrogen by Cu ions in zeolites. As a result, zeolites with copper were used for the one-pot production of dimethylether by hydrogenation of CO and CO_2_ over hybrid catalysts: CuZnAl/ZSM-5 and CuZrZn/ferrierite [43,44].

## 2. Results and Discussion

### 2.1. Structure of the Studied Catalysts

**Figure 2:**
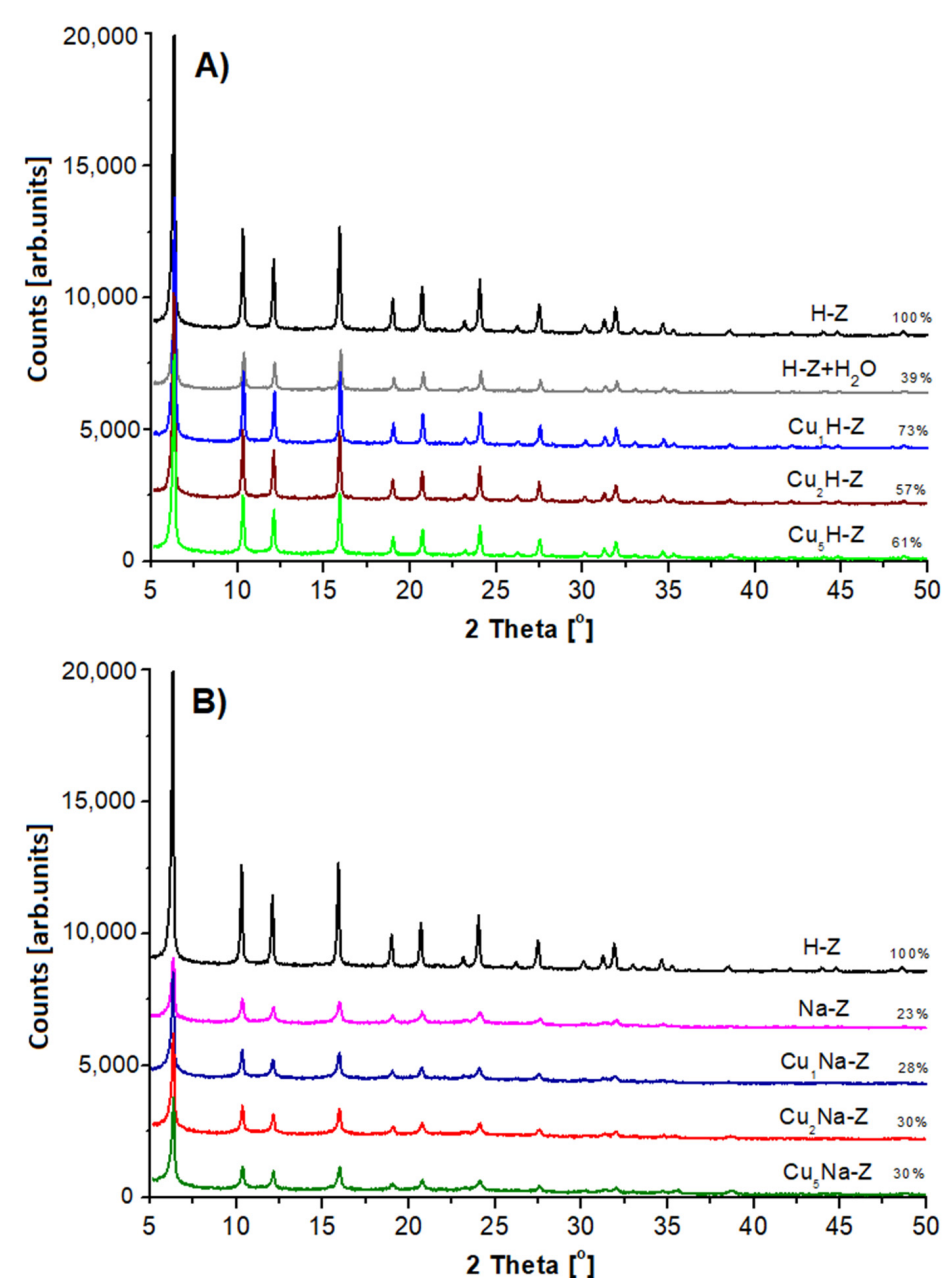
Powder XRD patterns of modified zeolites: (**A**) samples modified only with Cu(NO_3_)_2_·3H_2_O; (**B**) samples pretreated with NaNO_3_ and then modified with Cu(NO_3_)_2_·3H_2_O.

The X-ray diffraction (XRD) patterns collected for the studied catalysts are presented in Figure 2. For all samples the presence of FAU type phase was found [45]. The way of the catalyst modification plays an important role in the crystallinity of the final form of the samples. FAU31-based catalysts impregnated with Cu solution (Cu_x_H-Z) are characterized by lower crystallinity (57–73%) in respect to the parent sample (H-Z-100%). The crystallinity loss depended weakly on the amount of the introduced copper.

Another effect was observed in case of the samples pretreated with sodium nitrate series (Na-Z). The application of the procedure referring to the removal of Brønsted acid sites with NaNO_3_ (pH = 7) led to a significant deterioration of zeolite crystallinity (from 100 to 23%). Further treatment with copper (Cu_x_Na-Z) leads to no apparent changes in the crystallinity of the samples (28–30%).

The explanation of the observed effect seems to be complex. At first sight, observed phenomenon could be explained as a result of hydrolysis of Si-O-Al groups occurring in zeolites [46]. H-Z treated with a pure distilled water (at the same pH and temperature) subjected to a distinct decrease of crystallinity (from 100 to 39%). On the other hand, it is worth emphasizing that the partially dealuminated H-Z (used here as a parent sample) has a fractured structure, which is more sensitive to any modification in relation to the virgin FAU, as shown by Gackowski et al. [17,47]. That leads to the amorphization of the “nibbled” FAU-type zeolite.

Observed differences in the crystallinity loss of the final form of prepared samples depending on the chemical composition of used mixture for H-Z modification might be associated with an ionic strength of the employed aqueous solutions. Further research concerning this matter will be continued.

For all Cu-zeolites (both Cu_x_H-Z and Cu_x_Na-Z), no XRD patterns of copper phase were found. This indicates that the incorporated copper forms crystals, which can be too difficult for identification by this technique.

**Figure 3:**
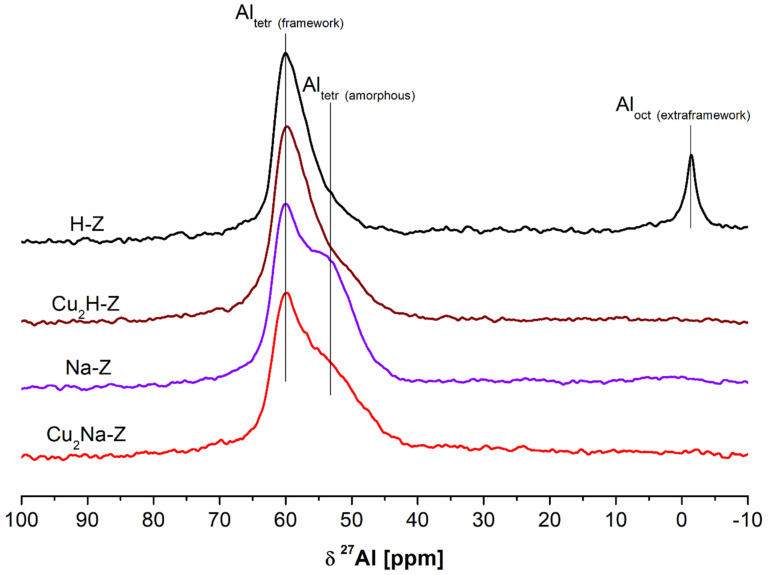
^27^Al NMR spectra of different prepared samples.

^27^Al MAS NMR spectra of the parent and modified samples are visualized in Figure 3. The employed modification procedures have an influence on the status of aluminum.

Spectrum of the parent H-Z shows two signals: at 61.4 ppm related to “zeolitic” tetrahedral Al and at 0 ppm associated with octahedrally coordinated extraframework Al.

Treatment of H-Z either with NaNO_3_ or Cu(NO_3_)_2_·3H_2_O leads to the disappearance of the signal at 0 ppm coming from extraframewok Al. Simultaneously, a new signal at ca. −53 ppm appeared. The signal is typical of tetrahedral (non-zeolitic) Al present in amorphous aluminosilicates, which was also reported by Gackowski et al. [17] and Kuterasiński et al. [48]. This signal is particularly strong in the spectra of the sample modified with NaNO_3_ alone (Na-Z), for which the most noticeable decrease of crystallinity was found. Hence, it may be concluded that the contribution of this type of aluminum species increases with the amorphization of parent H-Z zeolite [49]. Another possibility is the change of the status of aluminum as a result of the interaction between Al species coming from zeolitic structure and extraframework cations originating from modifiers, such as pyridine, ammonia and salts (Na^+^ or K^+^), which was reported by Bourgeat et al. [50]. Based on the data summarized in [50], we may conclude that similar situation in the case of the treatment of our FAU-type zeolite with aqueous Cu(NO_3_)_2_ solution takes place.

### 2.2. Porosity of the Prepared Catalysts

The porosity of the studied samples is presented in Table 1. The parent H-Z zeolite contained both micropores (the volume of which was 0.26 cm^3^/g) and mesopores (0.29 cm^3^/g). The external surface area and average pore diameter were 278 m^2^/g and 29.0 Å, respectively.

The choice of the post-synthesis modification had a pronounced effect on the porous structure of the studied samples. The direct modification of H-Z with Cu (Cu_x_H-Z) causes slight increase of external surface area from 278 to 280–296 m^2^/g and small loss of the micropore volume from 0.26 to 0.21 cm^3^/g. Neither the volume of mesopores nor average pore diameter were changed.

The utilization of NaNO_3_ to obtain the sodium form of H-Z (Na-Z) led to significant loss of microporous structure. The volume of micropores decreased from 0.26 to 0.15 cm^3^/g with a simultaneous increase of mesopores volume from 0.29 to 0.59 cm^3^/g. The external surface area and average pore diameter increased from 278 to 529 m^2^/g and from 29.0 to 36.7 Å, respectively. The observed changes in the porosity of Na-Z in relation to parent H-Z may result from the destruction of microporosity and the simultaneous production of larger mesoporous pores.

Further treatment with aqueous copper nitrate solution leads to small changes in the porous structure of such modified samples (Cu_x_Na-Z). Both an external surface area and the volume of mesopores for Cu-containing samples were smaller in comparison with Na-Z: (443–459 m^2^/g vs. 529 m^2^/g and 0.52–0.55 cm^3^/g vs. 0.59 cm^3^/g, respectively).

### 2.3. Morphology of the Studied Samples

**Figure 4:**
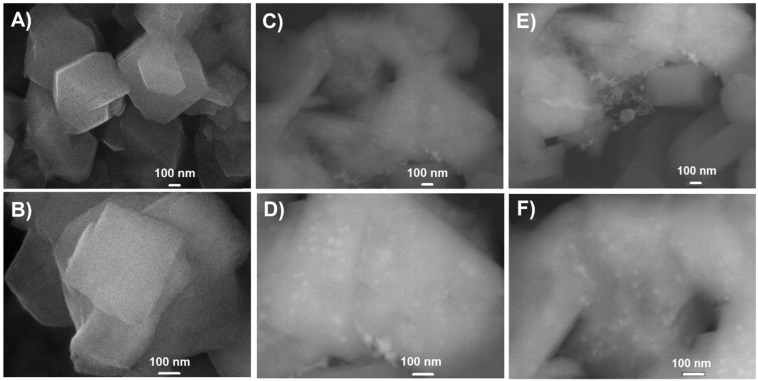
SEM images of the chosen samples: H-Z as a parent sample: (**A**) 50,000 x, (**B**) 100,000 x; Cu_5_H-Z sample: (**C**) 50,000 x, (**D**) 100,000 x; Cu_5_Na-Z sample: (**E**) 50,000 x, (**F**) 100,000 x.

The scanning electron micrographs of the variously modified samples are presented in Figure 4 and TEM images are additionally included in Appendix A Figure A5. The FAU31-based catalyst particles are of irregular shape. However, in the case of parent sample (H-Z) grains are prisms and are characterized by sharp and clear edges. Either for the sample directly modified with Cu species (Cu_5_H-Z) or pretreated with NaNO_3_ and then modified with Cu(NO_3_)_2_ (Cu_5_Na-Z), some amorphization took place. That resulted in the blurring of the prism-shaped crystals with sharp edges into streamlined agglomerates of amorphized zeolite grains. From SEM micrographs, it may be concluded that copper active phase is generally well distributed over a zeolitic carrier. Only a very small part of copper formed clusters. That is in line with XRD images (Figure 2), for which no additional reflexes attributed to copper phase were observed.

Table 1 presents the spatial distribution of selected elements: Si, Al, O and Cu for all prepared samples. The EDS analysis leads to the conclusion that the treatment of parent zeolite (H-Z) with either NaNO_3_ or Cu(NO_3_)_2_ did not cause relevant changes in chemical composition of bare zeolitic carrier. The amounts of Si, Al and O were in the ranges of 23.3–27.7%wt., 0.6–1.6%wt. and 67.2–75.8%wt., respectively. When the copper was taken into account in our considerations, it was found that copper content detected by EDS depended on the way of samples preparation (Cu_x_H-Z vs. Cu_x_Na-Z). For protonic series, the amount of copper found by EDS increased with the real amount of copper introduced into FAU-type zeolite by the impregnation procedure. Comparison of the coper contents from EDS and really introduced by the impregnation (0.9%wt. or 2.6%wt. or 3.1%wt. vs. 1.0%wt. or 2.0%wt. and 5.0%wt., respectively) led to the conclusion that the great majority of copper was located on the surface zeolitic grains. Another situation took place in the case of sodium series: Copper content found by EDS depended weakly on the real amount of Cu deposited on the zeolite during the impregnation and was in the range of 1.0–1.5%wt. Hence, particularly for the sample containing 5%wt. of copper (Cu_5_Na-Z), most of copper was located within such modified zeolitic grains and was inaccessible for the detection by EDS technique.

### 2.4. Reducibility of the Catalysts

**Figure 5:**
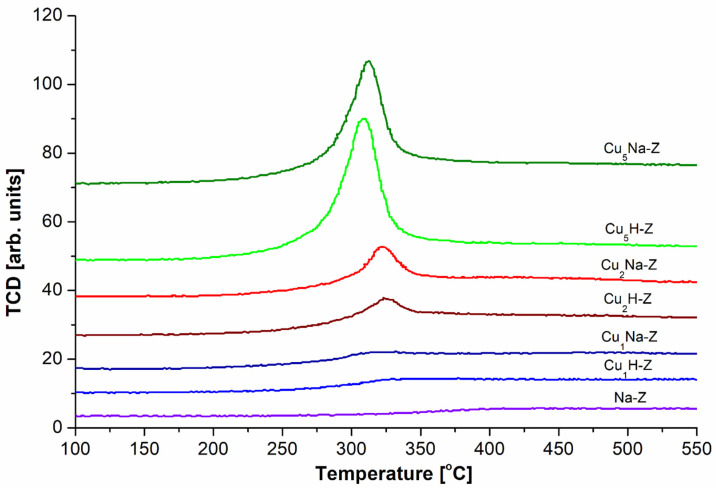
H_2_-TPR profiles for Cu-containing zeolites.

Figure 5 shows H_2_-TPR profiles of the prepared samples. Generally, the size and the position of the reduction peaks depended both on the copper content and on the preparation method of the catalyst. No reduction peaks were found for the catalysts with 1%wt. of Cu probably due to too low amount of the active phase. For the samples with 2%wt. of Cu, pretreatment with sodium nitrate and subsequent impregnation with Cu (Cu_2_Na-Z) resulted in the occurrence of the reduction peak with the maximum at the similar temperature (321 °C) in relation to the sample directly modified with copper nitrate (Cu_2_H-Z), for which maximum was found at 325 °C. Further increase of the amount of Cu in the samples resulted in the appearance of bigger reduction peaks. The way of modification of the studied samples had not the influence on the shape of the reduction peaks. In the case of Cu_5_H-Z, the obtained signal with maximum at 310 °C was greater than the signal registered for Cu_5_Na-Z with maximum localized at 320 °C. In all cases the obtained wide reduction peaks may be assigned to Cu^2+^→Cu^0^ [51,52,53,54,55].

### 2.5. Character of the Active Sites as Evidenced by IR Spectroscopy with CO and NH_3_ as Probe Molecules

**Figure 6:**
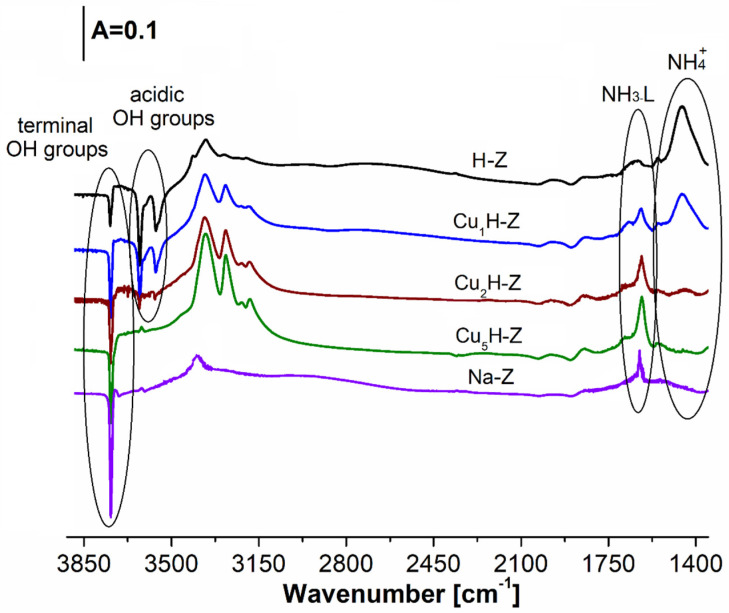
The co-existence of Brønsted and Lewis acid sites in dependence on the modification procedure of catalysts.

Figure 6 shows the differential IR spectra recorded for the samples activated at 400 °C and after adsorption of ammonia at 130 °C. In the case of H-Z, Cu_1_H-Z and Cu_2_H-Z, the presence of Brønsted acid sites was evidenced. Raising Cu content causes a decrease of the intensity of the bands at 3620, 3550 and 1450 cm^−1^ assigned to the acidic OH groups from the zeolite structure. That implies from ionic-exchange of protons by copper cations. Simultaneously, the increase of 1620 cm^−1^ band intensity related to Lewis acid sites was found. The occurrence of Lewis acid sites originated both from aluminum in FAU31-type zeolite as well as from the copper species [56,57,58]. In this case, the introduction of copper species into FAU31-zeolite structure is responsible for the increase of Lewis acidity. For H-Z, Cu_1_H-Z, Cu_2_H-Z and Cu_5_H-Z, concentrations of Brønsted and Lewis acid sites formed the following sequences: (160 vs. 90 μmol/g), (80 vs. 211 μmol/g), (20 vs. 314 μmol/g) and (0 vs. 441 μmol/g), respectively (Table 1). (Cu_x_H-Z). In all cases the presence of terminal OH group was detected (see bands at 3740 cm^−1^). The sample treated with NaNO_3_ (Na-Z) was deprived of protonic acidity—no bands at 3620, 3550 and 1450 cm^−1^ were found.

**Figure 7:**
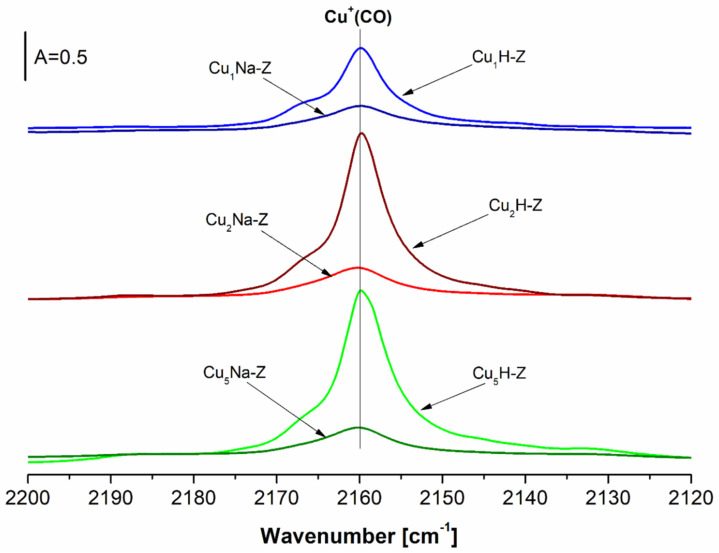
IR spectra of CO adsorbed at 25 °C on the surface of Cu-zeolite samples.

The results from the differential IR spectra and the quantitative analysis obtained from the experiments with the CO adsorption on the Cu-containing samples at room temperature are reported in Figure 7 and Table 1. For the samples directly impregnated with copper (Cu_x_H-Z), the increasing Cu content from 1%wt. to 2%wt. led to much higher concentration of Cu^+^ (from 75 to 160 μmol/g). Further rising of the amount of copper up to 5%wt. had no additional effect (160 vs. 170 μmol/g). It may be concluded that the cationic exchange positions were filled fully by Cu^+^ present in high amount in the FAU-based samples.

The data obtained for the zeolites pretreated with NaNO_3_ and then modified with Cu (Cu_x_Na-Z) indicated low concentration of Cu^+^ (ca 30 μmol/g), independent on the copper content in the studied samples. The distinct amorphization of the zeolitic structures led to the drastic deterioration of exchange capacity of such modified catalysts.

The presence of Cu^+^ in our samples subjected to FT-IR studies originates from the so-called “auto-reduction” of Cu^2+^ species in vacuum at high temperature (400 °C and higher) in the absence of a reducing agent. This phenomenon takes place during the activation procedure prior to the FT-IR measurements of our Cu-samples with adsorbed probe molecules. The auto-reduction mechanism of divalent copper species was previously described for Cu-loaded both zeolites [59] and alumina [60]. For comparison, in the H_2_-TPR results (Figure 5), we observed direct reduction of Cu^2+^ species to Cu^0^ (without the formation of Cu^+^ which is generally difficult to keep stable). This ostensible contradiction between TPR and FT-IR data is due to different pretreatment conditions. In the former case, an activation prior to the H_2_-TPR experiment was performed in the absence of vacuum (in a helium flow) at much lower temperature compared to FT-IR measurements (100 °C vs. 400 °C in vacuum). Hence, during the activation preceding H_2_-TPR experiment, the auto-reduction of divalent Cu species did not take place.

In our previous work [61], we reported that for the sample in protonic form and containing 1%wt. of Cu (Cu_1_H-Z), the concentration of Cu^+^ was twice smaller compared to the Cu content originating from its introduction by the impregnation method (160 μmol/g). This phenomenon may be explained by the fact that in our zeolite (FAU31), Cu^+^ may be located both in supercages and inside hexagonal prisms and cuboctahedra. The latter location of cations makes them inaccessible to adsorbed particles, such as CO. The “hidden” sites are occupied by cations in the first place, due to the highest effectiveness of the stabilization of cations by framework oxygens in these positions.

In the case of samples containing higher Cu contents (2 and 5%wt. both for protonic and sodium series), lower amount of Cu interacting with CO in relation to the total copper content introduced during impregnation may be also explained by the status of Cu species found in our FAU-31-based samples. It was indicated that the copper was in the form both of cations in exchange positions (Cu^+^_exch_, Cu^2+^_exch_.) and in the form of oxides (Cu^+^_oxide,_ CuO). Zeolites in sodium form (Cu_x_Na-Z) were characterized by much lower Cu^+^_exch_. contents and higher amounts of Cu^+^ and Cu^2+^ in the oxide forms [61].

**Figure 8:**
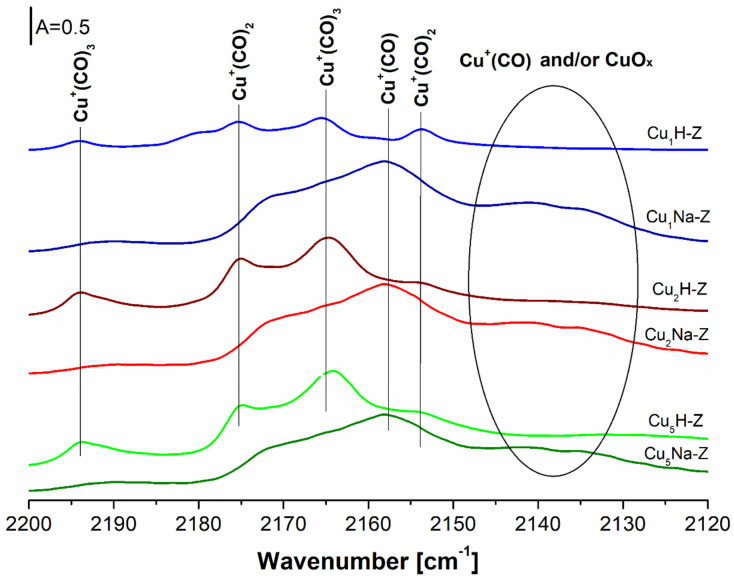
IR spectra of CO adsorbed at −100 °C on the surface of Cu-zeolite samples.

The differential IR spectra of the CO adsorbed at −100 °C on Cu-zeolite samples (Cu_x_H-Z) are presented in Figure 8. The adsorption of CO resulted in the appearance of the following bands: ν_asym_ at 2153 cm^−1^ and ν_sym_ at 2175 cm^−^^1^, corresponding to dicarbonyls Cu^+^(CO)_2_ formed at higher CO loadings [62,63]. The occurrence of new bands at 2165 and 2193 cm^−1^ assigned to tricarbonyls Cu^+^(CO)_3_ was also found [64,65]. The intensity of the mentioned bands increases along with the increased Cu content in a similar way as in the case of bands at 2160 cm^−1^ corresponding to Cu^+^(CO), which were recorded at room temperature (Figure 7).

For the samples additionally pretreated with NaNO_3_ (Cu_x_Na-Z), the bands at 2158, 2135 and 2140 cm^−1^ were registered (Figure 8). While the band at 2158 cm^−1^ was assigned to Cu^+^(CO), the interpretation of the bands at 2135 and at 2140 cm^−1^ was not straightforward. They may be assigned to another kind of copper site [66] or to the associated Cu ions [67]. Datka et al. [62] evidenced simultaneous occurrence of species by the presence of the bands at 2158 and 2135 cm^−1^ which allowed us to suppose that the adsorption of CO was not an equilibrium process at low temperature (−100 °C). CO interacted with copper by adsorbing on all accessible sites. Subsequently, the redistribution took place: CO desorbed from sites of lower adsorption heat and re-adsorbed on sites having higher binding energy. Due to the low rate of desorption at −100 °C (requiring CO molecules to overcome the activation energy), the redistribution was also slow. Góra-Marek et al. [68] suggested that the bands at ca 2130 cm^−1^ may correspond to CuO_x_ species.

In all cases (Cu_x_H-Z, Cu_x_Na-Z), the absence of the bands at 2206 and 2124 cm^−1^ proves that neither Cu^0^ nor Cu^2+^ species were present in the studied systems.

In our previous work [69], we reported the FT-IR analysis of Cu containing FAU31 zeolites in order to investigate the process of reduction of Cu sites with hydrogen and the properties of reduced zeolites. IR spectra of OH groups for two types of Cu-zeolites: in protonic (Cu_x_H-Z) and sodium form (Cu_x_Na-Z) were recorded. In all cases, the reduction of Cu sites with hydrogen resulted in the formation of Brønsted acid sites—the growth of IR bands of acidic hydroxyls was observed. Furthermore, quantitative analysis (NH_3_ sorption) indicated the production of protonic acidity, which agreed with the fact that OH bands increased. The formation mechanism of protonic acidity may be explained by the reduction of copper species (Cu^+^_exch_, Cu^+^_oxide_, Cu^2+^) with hydrogen according to the reactions, as follows: 2 Cu^+^ + H_2_ = 2 Cu^0^ + 2H^+^ and Cu^2+^ + H_2_ = Cu^0^ + 2H^+^. Furthermore, based on FT-IR studies of CO and NO sorption over Cu-FAU31 zeolites, it was indicated that the susceptibility of Cu sites for the reduction with hydrogen depended strictly on the type of Cu species. Order of reduction Cu sites formed the following sequence: Cu^+^_oxide_ > Cu^2+^ > Cu^+^_exch_ [69].

### 2.6. Catalytic Properties

Table 2 gathers the results of the catalytic tests for all four types of the samples: H-Z containing only Brønsted and Lewis acid sites coming from zeolite, Cu_2_H-Z containing both protonic and Lewis acid sites including also copper redox active centers, Na-Z samples possessing only Lewis acid sites originating from zeolite structure and Cu_2_Na-Z containing Lewis acid sites from both zeolite and copper species.

The data obtained at 300 °C (Table 2) indicated that Brønsted acid sites and copper active sites were similarly responsible for the catalytic activity of the studied materials. The best catalytic properties were found for the system containing both protonic and copper active sites (Cu_2_H-Z), for which the conversion of furfural was 73%, while furan yield was 22%. Somewhat worse catalytic properties were observed for the sample containing only acid sites attributed to zeolitic structure (H-Z). Conversion of furfural and yield of furan were 33% and 16%, respectively. In the case of the “Brønsted -free” catalyst (Na-Z) no catalytic activity was found, which evidenced no catalytic participation of Lewis acid sites assigned to Al species in this reaction. In turn, the presence of the copper redox sites in sodium form of FAU31-type zeolite (Cu_2_Na-Z) resulted in an apparent furfural conversion (53%), but very low furan yield (10%) in comparison with protonic counterpart (Cu_2_H-Z).

The increase of temperature up to 400 °C resulted in a significant improvement of catalytic properties of the parent sample (H-Z), for which furfural conversion and furan yield rose from 33 to 77% and from 16 to 60%, respectively. For the catalyst containing both protonic acidity and copper active sites (Cu_2_H-Z), the conversion of furfural decreased slightly from 73 to 64% and furan yield rose from slightly from 22 to 28%, respectively. When the reaction was carried out over Cu_2_Na-Z catalyst, higher temperature led to a very slight increase of both the conversion of furfural and furan yield (from 53 to 56% and from 10 to 14%, respectively). At 400 °C, no catalytic activity was found for the sodium sample (Na-Z).

Next, the influence of the amount of copper on the catalytic activity was checked—See Table 3 and Table 4. For the catalysts containing both protonic acid sites and copper active centers (Cu_x_H-Z—Table 3), the appearance of copper in FAU31 zeolite led to increase of both conversion of furfural (from 33 to 73–78%) and yield of furan (from 16 to 18–36%) at 300 °C. Nevertheless, the increase of the copper content led to significant deterioration in furan yield from 36% (Cu_1_H-Z) to 18% (Cu_5_H-Z).

Elevation of reaction temperature up to 400 °C resulted in a slight decrease of furfural conversion from 73–78% to 64% and some changes in furan yield: from 36 to 23%, from 22 to 28%, and from 18 to 19% for Cu_1_H-Z Cu_2_H-Z and Cu_5_H-Z, respectively. For the samples with higher copper content (Cu_2_H-Z and Cu_5_H-Z), trace of 2-methylfuran yield was found (1%).

In the case of catalysts deprived of Brønsted acid sites (Cu_x_Na-Z), studied at 300 °C, the increase of the copper content from 1 to 5%wt. resulted in a significant improvement of furfural conversion (47 vs. 71%) without relevant changes of furan yield (7–10%—see Table 4). Furthermore, rising copper loading caused increasing (but still very low) of the 2-methylfuran yield (from 2 to 4%).

At 400 °C, the increase of copper loading from 1%wt. to 5%wt. led to higher furfural conversion from 21 to 60%, but with very poor production of furan (5–14%). Another effect of higher copper content was increasing yield of 2-methylfuran (from 1 to 3%).

The application of the Cu-containing catalysts implies from their lower hydrogenation ability compared with noble metals, which allows it to avoid the hydrogenation of furan ring [70,71]. The nature of the active species has been a subject of discussion for many years [72]. It was found that the Cu^+^/Cu^0^ ratio in the catalyst decides about catalytic activity. Rao et al. [73] indicated that the turnover frequency (TOF) decreased with lowering Cu^+^/Cu^0^ ratio from 0.3 to 0 over carbon-supported Cu catalysts. This phenomenon may be explained by the activation of H_2_ by metallic copper species, while Cu^+^ sites plays a role of electrophilic or Lewis acid sites able to polarize C=O bond via electron lone pair of oxygen [74,75]. The Cu^+^/Cu^0^ ratio depends strongly on many factors, such as the kind of support, the way of preparation and the conditions of catalytic tests (including reduction meant as a pretreatment procedure).

In our previous work [61], we reported the status and properties of Cu species in HFAU and NaFAU of Si/Al = 31 by IR spectroscopy with CO and NO as probe molecules. Both Cu^+^ and Cu^2+^ were found in the zeolites in the form both of exchange cations (Cu^+^_exch_, Cu^2+^_exch_._)_ and in oxide form (Cu^+^_oxide_, CuO). The proportion between various forms of Cu depended significantly on both the amount of Cu introduced into the materials and the way of treatment of FAU-type zeolite (HFAU or NaFAU). In CuHFAU containing 1wt.% of Cu, all of Cu species were in the form of Cu^+^_exch_. neutralizing AlO_4_^-^. Higher copper content resulted in the co-existence of Cu^+^_oxide_ as well as the presence of Cu^2+^ as Cu^2+^_exch_. and CuO species. Zeolites Cu_x_NaFAU contained much lower amount of Cu^+^_exch_. and higher contribution of Cu^+^ and Cu^2+^ in the oxide forms. Direct comparison of the data given in Table 4 led to the conclusion that Cu^+^_oxide_ and CuO are the most active Cu species in this reaction. Furthermore, copper oxide species favor slightly the formation of 2-methylfuran.

The results of the catalytic experiments described above indicate that two types of active sites present in the tested samples contribute to their catalytic activity. Decarbonylation of furfural is realized mainly on the protonic acid sites.

High activity of the H-Z sample in furfural decarbonylation should be attributed to the existence of strong Brønsted acid sites. The similar reactivity of samples of acidic character was reported earlier for catalysts based on zeolites and heteropolyacids—see, e.g., references [76,77,78] for reports on transformation of lactic acid and its derivatives to acetaldehyde. The activity in decarbonylation was ascribed to high density of acid sites which directly interact with the carbonyl group which is to be removed from the substrate.

Furfural decarbonylation to furan also proceeds on Cu sites.

Our findings are in line with the previous reports on the application of the copper-based systems to furfural hydrogenation and decarbonylation [79]. A similar effect was also found for γ-valerolactone valorization [80]. It is claimed that furfural can be activated on copper phase via C-H bond rupture from the aldehyde group. Thus, formed furfuryl residue is stabilized on the metal center due to its interactions with un-filled orbitals of copper. The reactivity of furfural is also determined by the geometry of the substrate adsorption on the catalytic active sites [72].

Further, it is believed that the copper centers are also responsible for hydrogen activation during hydrogenation of biomass-derived products [80,81].

The contribution of copper redox active centers in the decarbonylation of furfural may be also explained by the production of Brønsted acid sites as a result the reduction of copper species with hydrogen according to the following reactions: 2 Cu^+^ + H_2_ = 2 Cu^0^ + 2H^+^ and Cu^2+^ + H_2_ = Cu^0^ + 2H^+^, which was reported in our previous work [69]. Protonic acid sites generated in this way could participate in the formation of furan.

We also investigated an effect of the application of the pretreatment in hydrogen (prior to catalytic experiment) on the catalytic behavior of the samples containing various copper content in protonic form—Cu_x_H-Z (Table 5). It was indicated that for Cu-containing catalysts, the use of hydrogen did not change furfural conversion (64%), but caused a slight increase of furan yield from 19 to 23% or from 17 to 28% or from 11 to 19% for Cu_1_H-Z or Cu_2_H-Z or Cu_5_H-Z, respectively. Analysis of these results suggests that the pretreatment of our catalysts in hydrogen (directly before catalytic measurements) led to the formation of new protonic acid sites, which could enhance the production of furan.

All studied catalysts revealed very high susceptibility to coking (Table 2, Table 3, Table 4 and Table 5). This effect was particularly strong for Cu-containing catalysts and generally increased with the amount of copper in the prepared samples. Deactivation is the main problem for Cu-based hydrogenation catalysts, which hampers their industrial application. This phenomenon also occurs over the catalysts containing acid sites like zeolites [81]. Hence, it is possible that newly created protonic acid sites take part in the production of coke. It may be also related with the loss of the Cu^+^ species by the reduction and poisoning of active Cu sites by the adsorption of reaction intermediates.

**Figure A1:**
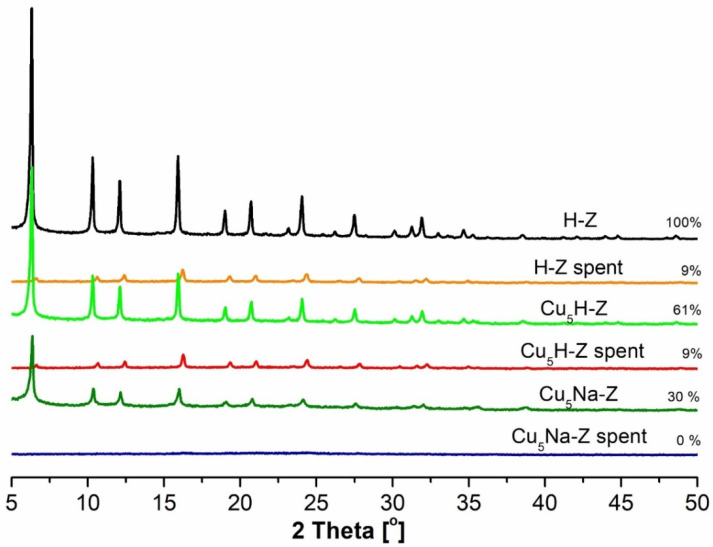
XRD images of the chosen samples before and after catalytic studies.

**Figure A2:**
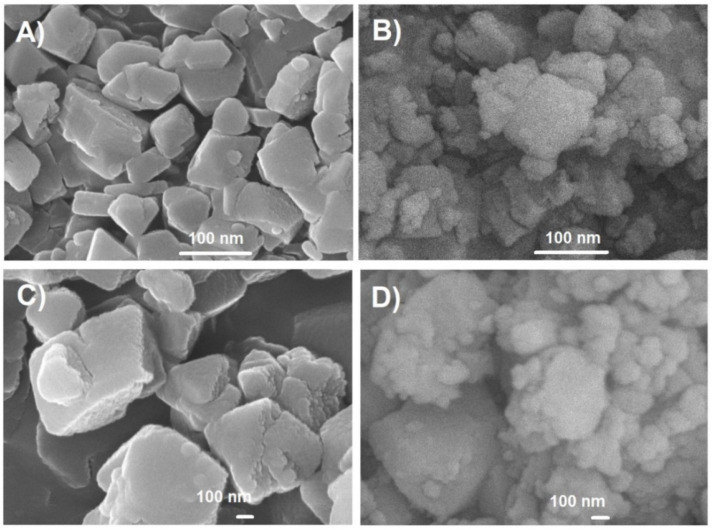
SEM images of the parent sample: fresh H-Z: (**A**) 25,000 x, (**B**) 50,000 x; spent H-Z: (**C**) 25,000 x, (**D**) 50,000 x.

**Figure A3:**
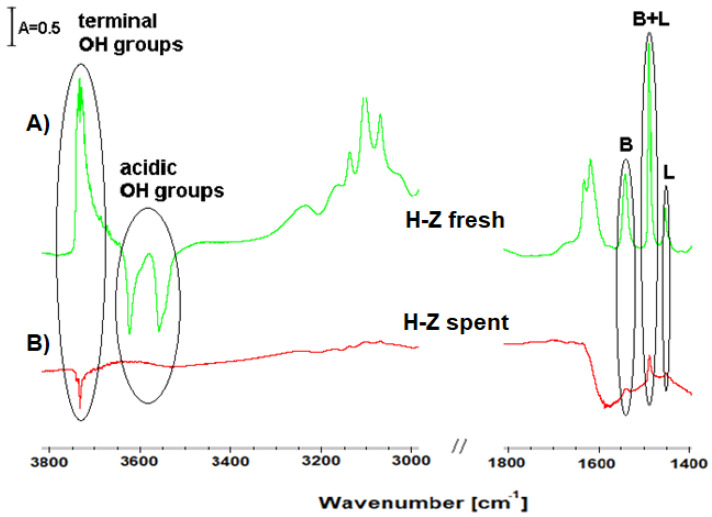
FT-IR spectra of the H-Z catalyst: (**A**) fresh sample, (**B**) spent sample (after reaction).

**Figure A4:**
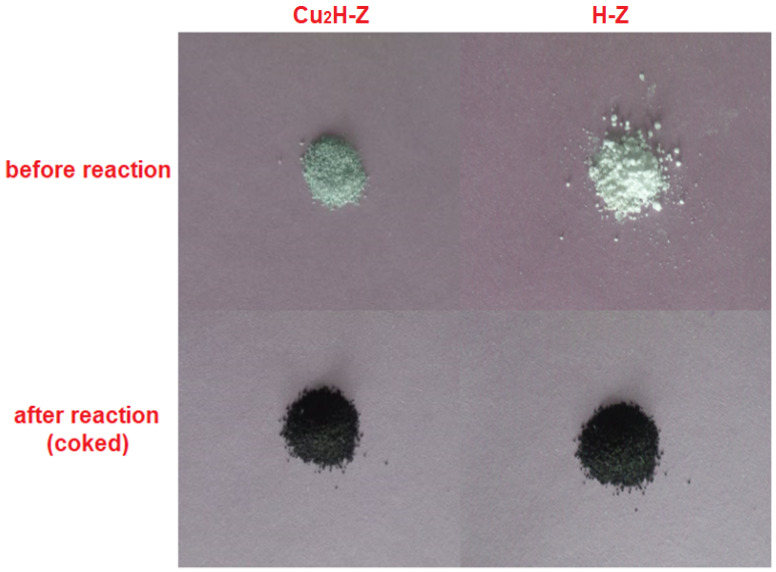
An optical evidence for the coking of the investigated samples during catalytic experiments.

**Figure A5:**
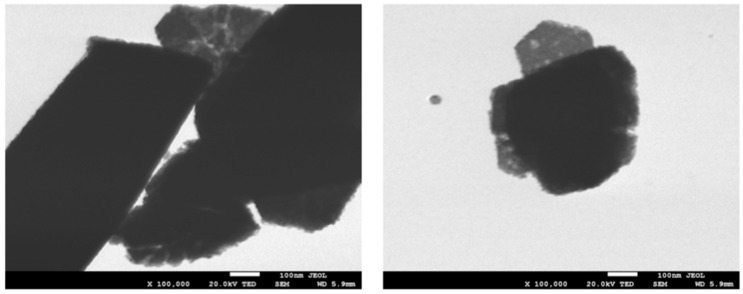
TEM images of the H-Z sample.

In order to determine how the studied catalysts changed after the reaction, we performed XRD, SEM and FT-IR experiments for the spent samples. The results concerning the crystallinity, morphology, acidic properties and an optical appearance of the catalysts after catalytic experiments are summarized in Appendix A (Figure A1, Figure A2, Figure A3 and Figure A4).

In all cases, the analysis of XRD data (Figure A1) indicated that, during catalytic tests, the crystallinity of the studied samples underwent a drastic deterioration probably due to the interaction between vaporized furfural and very a sensitive structure of FAU31-type zeolite. Final crystallinity was ca 9% for the samples in protonic series (H-Z and Cu_5_H-Z), while Cu_5_Na-Z was characterized by zero crystallinity.

For the parent sample (H-Z), the contact with the reaction mixture led to apparent changes in its morphology (Figure A2). Well defined edges of irregular-shaped crystals were subjected to blurring, which could be an effect of the FAU31 amorphization.

Comparison of FT-IR spectra of pyridine adsorbed on fresh and spent H-Z catalyst led to the conclusion that studies catalyst underwent coking. The decrease of the bands assigned to the acid sites was probably due to blocking of acid sites by coke depositions.

Last evidence for the coking of the studied catalysts is a direct comparison of the optical appearance of samples before and after catalytic experiment (Figure A4). Both Cu-free and samples containing Cu became black during catalytic tests.

For the H-Z catalyst, a stability test was performed at 350 °C (Table A1). From the presented data, it may be concluded that the investigated catalyst was not highly stable due to coking. Both furfural conversion and yield of furan decreased from 74 to 35% and from 61 to 26%, respectively. The deterioration of catalytic properties of the studied sample during the time of experiment was in line with the loss of Brønsted acid sites accessibility (Figure A3), as well as corresponded to the changes in the crystallinity (Figure A1) and morphology (Figure A2) of the studied catalyst.

## 3. Materials and Methods

### 3.1. Catalysts Preparation

We obtained four types of catalytic systems (Table 6). A commercial ultrastabilized and dealuminated zeolite with FAU type structure (molar Si/Al ratio was 31) from Zeolyst, denoted as H-Z, was applied as a carrier. The sample contains only the zeolitic acid centers.

Sodium form of FAU31 zeolite (designated as Na-Z) is deprived of protonic acid centers. It contains only Lewis acid sites coming from zeolite structure (Al species); thus, it may be used as a reference sample. It was obtained by 5-fold ion-exchange of H-Z with 0.5 M NaNO_3_ at 80 °C for 2 h. The mass of solution was 30-fold higher compared to zeolite. Subsequently, the sample was 4-fold centrifuged (RPM = 4000) and dried at 60 °C for 3 h.

Cu-containing Na-Z samples (denoted as Cu_1_Na-Z, Cu_2_Na-Z and Cu_5_Na-Z, where numerals mean %wt. of Cu) have both Lewis acid sites originating from both zeolite structure and redox active Cu centers. Na-Z was impregnated with 0.5 M Cu(NO_3_)_2_·3H_2_O, dried at 120 °C and, finally, calcined at 500 °C for 8 h.

In the case of samples for which pretreatment with NaNO_3_ was omitted (direct impregnation with aqueous copper nitrate solution under the same conditions as described above), all discussed active centers are present. The samples doped with 1, 2 and 5%wt. of Cu were denoted as Cu_1_H-Z, Cu_2_H-Z and Cu_5_H-Z, respectively.

### 3.2. Catalysts Characterization

The XRD measurements were carried out at room temperature using diffractometer PANalytical X’Pert PRO (Malvern Panalytical Ltd., Malvern, United Kingdom) MPD with CuKα radiation (λ = 1.5418 Å) at 40 kV and 30 mA and with 2θ range from 5 to 50° with 0.033° step for 12 min. The researched zeolite samples were in powder form and were placed in holders. The crystallinity changes of prepared samples were determined by the integration of the reflexes in the 2θ range of 5–50° in XRD patterns. XRD images are presented in Figure 2 and Figure A2 (in Appendix A).

The ^27^Al MAS NMR experiments were performed on hydrated zeolites by the exposition to the vapor of a saturated aqueous Mg(NO_3_)_2_ solution. The ^27^Al MAS NMR spectra were acquired at resonance frequency of 130.3 MHz, on a Bruker Advance III 500 MHz WB spectrometer (Bruker Corporation, Billerica, MA, USA). ^27^Al MAS NMR spectra were recorded at a spinning rate of the samples in zirconia rotors of 12 kHz with a short pulse length of 0.2 μs (π/12) and a recycle delay of 0.1 s. The chemical shifts were externally referenced to 1 M aqueous aluminum nitrate solution. ^27^Al MAS NMR spectra are given in Figure 3.

The low-temperature sorption of nitrogen was carried out using Quantachrome Nova 2000 (Quantachrome Instruments, now Anton Paar GmbH, Graz, Austria). Prior to the experiment, the samples were evacuated for 20 h at 300 °C in vacuum. Micropore volume (V_micro_) and external surface Area (S_EXT_) were calculated using t-plot method. Pore size distribution and micro-and mesopores volume (V_micro_, V_meso_) were determined by the utilization of BJH model to the adsorption branch of the isotherm. The results of porous structure measurements are summarized in Table 1.

The SEM experiments were carried out using Jeol JSM-7500F scanning electron microscope (JEOL Ltd., Tokyo, Japan) equipped with the X-ray energy dispersive (EDS) system INCAPentaFETx3 (JEOL Ltd., Tokyo, Japan). Two detectors were used and the images were recorded in two modes; the secondary electron detector provided SEI images and backscattered electron detector provided BSE (COMPO) micrographs. The samples were dried for 24 h and coated with chromium (20 nm) directly before measurements. SEM micrographs are given in Figure 4 and Figure A2 (in Appendix A).

The TPR profiles of the catalysts were obtained using U-type quartz reactor with a Quantachrome Chembet-300 apparatus (Quantachrome Instruments, now Anton Paar GmbH, Graz, Austria). All samples (25 mg each) were activated in helium flow at 100 °C for 1.5 h. Subsequently, the samples were heated (at 10 °C/min.) in the flow of reduction mixture (5% of H_2_ in Ar) to the final temperature 600 °C. The hydrogen consumption was monitored using TCD detector. The results referring to the reducibility of copper species in the studied catalysts are depicted in Figure 5.

The FT-IR analysis was conducted using Nicolet 6700 spectrometer with MCT detector from Thermo Scientific (Thermo Fisher Scientific, Waltham, MA, USA). Before the FT-IR experiments, the samples in the form of thin wafers were activated for 1 h at 400 °C in vacuum. The CO or NH_3_ gases (Air Products 99.5% and 99.3%, respectively) and pyridine (Sigma-Aldrich Chemie GmbH, Taufkirchen, Germany, 99.8%) were used as adsorbates. Small doses of CO were adsorbed on the surface of sample both at −100 °C and at room temperature. Procedure of ammonia or pyridine adsorption was carried out at 130 or 170 °C, respectively. The amount of probe molecules introduced into the IR cell was calculated from the ideal gas law. The quantitative analyses of Brønsted and Lewis acidity were performed according to the procedures, which detailed description was reported in [17,48,68]. Either qualitative or quantitative analyses of the FT-IR analysis of adsorbed probe molecules are illustrated in Figure 6, Figure 7 and Figure 8 and Figure A3 (in Appendix A) and in Table 1.

### 3.3. Catalytic Tests

Catalytic studies of hydrogenation and decarbonylation of furfural to furan and 2-methylfuran were performed in a fixed-bed quartz microreactor. The experiments were done at atmospheric pressure and in the temperature range of 300–400 °C for 30 min at given temperature. For each experiment 0.5 mL of catalyst (particles sizes in the range of 400 to 500 microns) was placed on a quartz wool plug in the reactor (with an inner diameter of 8 mm and length of 32 cm) and reduced in a 25 mL/min flow of pure hydrogen at 400 °C for 2 h. Subsequently, it was applied a gaseous mixture containing 0.23 mg/min of furfural (SAFC, ≥98.0%) in H_2_ with total flow rate of 50 mL/min. WHSV equaled to 6000 h^−1^. The concentration of furfural, furan and 2-methylfuran as the main reaction products were continuously measured using gas chromatography SRI8610A model with FID detector SRI Instruments, Torrance, CA, USA).

The furfural conversion (X_FAL_), furan and 2-methylfuran yields (Y_F_, Y_2-MF_) were calculated according to the following equations:

X_FAL_ = (N_FAL_^in^ − N_FAL_^out^)/N_FAL_^in^ * 100% (1)

Y_F_ = N_F_^out^/(N_FAL_^in^ − N_FAL_^out^) * 100% (2)

Y_2-MF_ = N_2-MF_^out^/(N_FAL_^in^ − N_FAL_^out^) * 100% (3)

where N_FAL_^in^ and N_FAL_^out^ are moles of furfural before and after the reaction, respectively. In turn, N_F_^out^ and N_2-MF_^out^ are moles of products produced during the reaction: furan or 2-methylfuran, respectively. Prior to the catalytic experiments, blank tests including the contribution of quartz wool as well as a bare reactor on the catalytic behavior were per -formed. In the catalytic data summarized in Table 3, Table 4, Table 5 and Table 6, the effects attributed to blank tests have been taken account.

## 4. Conclusions

We have obtained the systems containing none, one or two types of active centers i.e., Brønsted acid sites and “redox” copper species. Treatment of parent FAU31 zeolite (H-Z) with NaNO_3_ aq. sol. (Na-Z) resulted in the removal of Brønsted acid sites.

Direct synthesis of furan from furfural was catalyzed both by copper-exchanged and copper-free dealuminated faujasite type zeolite. Brønsted acid sites were mainly responsible for the catalytic activity in the whole temperature range. No contribution of Lewis acid sites associated with zeolitic aluminum in catalytic activity was found. The presence of the redox copper centers led to the appearance of trace amounts of 2-methylfuran as the result of the partial hydrogenation of furfural. Coke formation was found, probably resulting from the polymerization of furfural. The presence of coper in the studied catalysts promoted coking. Further studies dedicated to the nature of furfural polymers will be done in the nearest future.

## Figures and Tables

**Table 1:** EDS (Energy Dispersive Spectroscopy) chemical analysis, N_2_ physisorption derived properties and the influence of Cu content on the concentration of active centers in the studied samples.

Sample	Chemical Analysis (EDS)				Porosity				Acidity		
	Si [%]	Al [%]	O [%]	Cu [%]	Si [%]	Al [%]	O [%]	Cu [%]	Si [%]	Al [%]	O [%]
**H-Z**	23.5	0.7	75.8	0	0.260	0.290	278	29.0	160	90	0
**Cu_1_H-Z**	27.3	1.1	70.7	0.9	0.218	0.292	280	29.9	80	211	75
**Cu_2_H-Z**	29.4	0.8	67.2	2.6	0.222	0.293	296	29.1	20	314	160
**Cu_5_H-Z**	26.7	0.6	69.6	3.1	0.213	0.283	289	28.9	0	441	170
**Na-Z**	24.4	0.9	73.7	0	0.154	0.594	529	36.7	0	249	0
**Cu_1_Na-Z**	23.1	1.3	73.4	1.0	0.126	0.546	458	38.8	0	n.m.	30
**Cu_2_Na-Z**	23.3	1.6	73.3	1.1	0.129	0.516	443	37.8	0	n.m.	30
**Cu_5_Na-Z**	27.7	1.4	68.5	1.5	0.131	0.536	459	38.0	0	n.m.	30

**Table 2:** Catalytic properties in dependence on reaction temperature and the presence of active center types in the studied samples.

Sample	Conversion [%]		Y_Furan_ [%]		Y_2-methylfuran_ [%]	
	300 °C	400 °C	300 °C	400 °C	300 °C	400 °C
H-Z	33	77	16	60	0	0
Cu_2_H-Z	73	64	22	28	1	1
Na-Z	0	0	0	0	0	0
Cu_2_Na-Z	53	56	10	14	2	2

**Table 3:** The influence of Cu content and temperature on catalytic properties of Brønsted acid sites-containing samples.

Sample	Conversion [%]		Y_Furan_ [%]		Y_2-methylfuran_ [%]	
	300 °C	400 °C	300 °C	400 °C	300 °C	400 °C
H-Z	33	77	16	60	0	0
Cu_1_H-Z	78	64	36	23	0	0
Cu_2_H-Z	73	64	22	28	1	1
Cu_5_H-Z	74	64	18	19	1	1

**Table 4:** The influence of Cu content and temperature on catalytic properties of Brønsted acid sites-free samples.

Sample	Conversion [%]		Y_Furan_ [%]		Y_2-methylfuran_ [%]	
	300 °C	400 °C	300 °C	400 °C	300 °C	400 °C
Na-Z	0	0	0	0	0	0
Cu_1_Na-Z	47	21	7	0	2	1
Cu_2_Na-Z	53	56	10	14	2	2
Cu_5_Na-Z	71	60	9	5	4	3

**Table 5:** The influence of the application of pretreatment in hydrogen at 400 °C on catalytic properties of Cu containing HFAU31-type samples. The presented catalytic results refer to 400 °C.

Sample	Conversion [%]		Y_Furan_ [%]		Y_2-methylfuran_ [%]	
Activation in H_2_	No	Yes	No	Yes	No	Yes
H-Z	-	77	-	60	-	0
Cu_1_H-Z	64	64	19	23	0	0
Cu_2_H-Z	64	64	17	28	3	1
Cu_5_H-Z	64	64	11	19	4	1

**Table 6:** The occurrence of different active center types in dependence on the preparation of the sample.

Sample	The Presence of	
	Brønsted	Copper Active Sites
H-Z	yes	no
Cu_x_H-Z	yes	yes
Na-Z	no	no
Cu_x_Na-Z	no	yes

x = 1, 2, 5% wt. Cu.

## Data Availability

Not applicable.

## Sample Availability

Samples of the compounds of Cu containing zeolites of FAU type structure are available from the authors.

## Appendix A

**Table A1:** Stability test for the H-Z catalyst at 350 °C.

Time [h]	Conversion [%]	Y_Furan_ [%]	Y_2-methylfuran_ [%]
0	74	61	0
1	55	42	0
2	44	36	0
3	39	29	0
4	38	27	0
5	36	27	0
6	36	26	0
7	35	26	0
